# Supplementary material for: Rapid Liquid Chromatography—Tandem Mass Spectrometry Analysis of Two Urinary Oxidative Stress Biomarkers: 8-oxodG and 8-isoprostane
Source: Antioxidants (Basel). 2020 Dec 31;10(1):38. doi: 10.3390/antiox10010038 (PMC7823279; doi:10.3390/antiox10010038)
Supplement: Supplementary file 1 [file antioxidants-10-00038-s001.pdf]

Supplementary Information

Rapid liquid chromatography – tandem mass spectrometry analysis of two urinary oxidative stress biomarkers: 8-oxodG and 8-isoprostane

Nicolas Sambiagio [1], Jean-Jacques Sauvain [1], Aurélie Berthet [1], Reto Auer [1,2], Anna Schoeni [2] and Nancy B. Hopf [1,*]

[1] Center for Primary Care and Public Health (Unisanté), University of Lausanne, Route de la Corniche 2, 1066 Epalinges-Lausanne, Switzerland; nicolas.sambiagio@unisante.ch; jean-jacques.sauvain@unisante.ch; aurelie.berthet@unisante.ch

[2] Institute of Primary Health Care (BIHAM), University of Bern, Mittelstrasse 43, 3012 Bern, Switzerland; reto.auer@biham.unibe.ch; anna.schoeni@biham.unibe.ch

* Correspondence: nancy.hopf@unisante.ch

This document contains the supplementary information referenced in the manuscript "Rapid liquid chromatography – tandem mass spectrometry analysis of two urinary oxidative stress biomarkers: 8-oxodG and 8-isoprostane" by Nicolas Sambiagio, Jean-Jacques Sauvain, Aurélie Berthet, Reto Auer, Anna Schoeni, and Nancy B. Hopf. This document contains seven pages with two Tables (S1-S2).

Description of the standard stock solutions

Stock solutions of 8-oxodG (1 mg/mL) and 8-isoprostane (1 mg/mL) in water/methanol (8:2) were stored at -20°C. Intermediate stock solutions of 8-oxodG (5 µg/mL) and 8-isoprostane (5 µg/mL) in water were prepared from the stock solution and stored at 4°C during the validation process. Internal standard (IS) stock solutions were made with [$^{15}N_5$]-8-oxodG (2.5 µg/mL) and 8-isoprostane-d_4 (2.5 µg/mL) in water and stored at 4°C during the validation process. Working solutions of 8-oxodG were freshly prepared at 100 ng/mL and 10 ng/mL in water, and 8-isoprostane at 100 ng/mL, 10 ng/mL and 1 ng/mL in water from the intermediate stock solutions before each sequence. Working solutions of [$^{15}N_5$]-8-oxodG (50 ng/mL) and 8-isoprostane-d_4 (25 ng/mL) in water were also freshly prepared before each sequence.

Description of the MS parameters (Table S1)

Table S1 – Multi-reaction monitoring parameters

**Compounds**	**Polarity**	**Mass transitions [m/z]**[1]	**Collision energy [V]**	**RF lens [V]**
8-oxodG	Positive	284.1 → 140.0	28.8	37
		**284.1 → 168.1**	10	37
		284.1 → 243.0	10.2	37
[$^{15}N_5$]-8-oxodG	Positive	**289.1 → 173.1**	10	40
8-isoprostane	Negative	**353.2 → 193.1**	25	80
		353.2 → 291.0	20	80
		353.2 → 309.2	20	80
8-isoprostane-d_4	Negative	**357.2 → 313.2**	25	78

[1] Mass transitions in bold are quantification transitions, others are confirmation transitions.

ESI parameters under positive detection mode (for 8-oxodG) were optimized at 3700 V, and under negative mode (for 8-isoprostane) at 3400 V. The vaporizer temperature was maintained at 350°C and the ion transfer tube at 390°C. The sheath gas and the auxiliary gas pressures were set at 45 and 17 Arb (arbitrary unit), respectively. The argon pressure was set at 1.5 mTorr.

Description of the method validation parameters

LODs were determined by injecting decreasing concentrations of analyte in water until obtaining a signal to noise ratio (S/N) of three, and LOQs until a signal to noise ratio (S/N) of ten. In urine, LOQs, corresponding to the lowest calibration points, were chosen according to the reported concentrations of the analyte. Criteria for linearity was a coefficient of determination R^2 greater than 0.999 for the urinary calibration curve. Intra-day precision and accuracy were determined from three replicates measurements of three concentrations with two different urine samples on the same day. Inter-day precision and accuracy were determined on three different days. The precision was expressed as the coefficient of variation. The accuracy was calculated as the ratio of the mean of the calculated concentrations of the spiked samples to the theoretical concentrations, and was expressed as a percentage. Extraction recovery was calculated by dividing the IS signal area of sample spiked before and after SPE, and was expressed as a percentage. Absolute matrix effects were calculated by dividing the IS signal area of sample spike after SPE and the IS signal area in spiked water without SPE, and was expressed as a percentage. Relative matrix effects were calculated by comparing slopes of calibration curves in three different urine samples and expressed as the coefficient of variation. Calibration curves in water and urine were also compared (ratio of the slope in water and in urine, expressed as a percentage). The stability of the compounds had been previously studied [1–3]. We investigated the stability of the analytes in urine after being frozen at -20°C for 6 months by analyzing QC aliquots (low and high) and monitoring the concentration changes along time. Twenty-one aliquots were analyzed over the 6-month period. We also investigated the stability of processed samples at room temperature (12 h) by injecting three times the same QC aliquot seven hours apart (on 21 different days).

Description of the optimization of the SPE (Table S2)

We investigated several SPE cartridges for the sample clean up and these are represented in Table S2. The two anion exchange SPE cartridges gave good recoveries for 8-oxodG but not for 8-isoprostane. During the tests with anion exchange cartridges, the samples were adjusted to basic pH ranges with ammonium hydroxide (0.05%). Chromabond C18 endcapped performed the best of the four other reversed-phase cartridges tested as the two analytes were well retained. We tested two different phase quantities (200 mg and 500 mg) and selected the bigger. We optimized the washing step and found that a small part of 8-oxodG (4 6%) was eluted during a washing step with 10% methanol. We chose a high volume of methanol (3 mL) for the elution to recover the total 8-isoprostane quantity.

Table S2. Summary of tested SPE cartridge during method development.

**SPE cartridge**	**Features**
EVOLUTE AX	Mixed-mode hydrophobic and strong anion exchange
CHROMABOND Easy	Polar modified polymer with weak anion exchange
Bond Elut C18 OH	Non-polar non-endcapped sorbent
Bond Elut NEXUS	Non-polar polymeric sorbent
ISOLUTE C18	Non-polar sorbent
CHROMABOND C18 ec	Non-polar endcapped sorbent

Comments on matrix effects observed in "dark urine"

"Matrix effects were also observed for "light urine" (67% for 8-oxo-dG and 83% for 8-isoprostane) and "dark urine" (4% for 8-oxodG and 25% for 8-isoprostane), estimated by the IS variation. A simple dilution by a factor two of "dark urine" reduced matrix effects to 19% for 8-oxodG and 58% for 8-isoprostane." It is important to mention that the dilution did not reduce the MS response of 8-oxodG and 8-isoprostane by two: 8-oxodG signal increased by 39% and 8-isoprostane decreased by only 21%.

We observed signal suppression due to matrix effects. This signal suppression was proportional to the concentration of the urine sample (i.e. the presence of co-eluting matrix components). This relationship was, however, not linear. This explains why we observed a signal increase after sample dilution. For example, the matrix effect for 8-oxodG ("dark urine") changed from 4% to 19% with a two-fold dilution. If we assume that the MS signal of the undiluted sample was 1, then the theoretical signal would be 25 (corresponding to 100%; no matrix effect). Diluting by two (and considering a linear response of the instrument with a slope of 1) then the theoretical signal of the two-fold diluted sample would be 12.5 (100%). Applying the matrix effect of 19% would give a signal of 2.4, which is effectively higher than the signal of the undiluted sample (1).

Signal suppression was different between the two analytes. There are more compounds co-eluting with 8-oxodG (4.7 min) than with 8-isoprostane (10.2 min). Generally, the closer the compounds are to the solvent elution (short retention times), the stronger the matrix effects. To counter balance this effect, appropriate internal standard are used for correcting for signal suppression. Stable isotopically labeled internal standards are preferred since their retention times are very close to those of the analytes and will undergo similar matrix effects as the biomarker.

Comments on matrix effects and method performance

The parameters that are directly impacted by matrix effects are LODs and LOQs. Indeed, several researchers report the LODs in aqueous solution (e.g. Wu et al. [2016]), some of them mentioning that this limit may vary in biological fluids (e.g. Martinez and Kannan [2018]) [1,4]. In reality, these parameters will change according to the urine samples, and they should be considered with precaution when assessing a method's performance. Calibration range, especially the lower calibration point, is more important as it delimits to which concentration the method is effective. However, analyte MS responses of samples must not be lower than the lower calibration point response, in which case they should be reinjected or reported as "under LOQ". This requires special attention because, due to matrix effects, a sample with the same concentration as the lowest calibration standard may have a lower MS response for the analyte and its internal standard (with the same analyte/IS ratio).

References

1. Martinez, M.P.; Kannan, K. Simultaneous Analysis of Seven Biomarkers of Oxidative Damage to Lipids, Proteins, and DNA in Urine. *Environ. Sci. Technol.* **2018**, *52*, 6647–6655, doi:10.1021/acs.est.8b00883.
2. Hu, C.-W.; Chao, M.-R.; Sie, C.-H. Urinary Analysis of 8-Oxo-7,8-Dihydroguanine and 8-Oxo-7,8-Dihydro-2′-Deoxyguanosine by Isotope-Dilution LC-MS/MS with Automated Solid-Phase Extraction: Study of 8-Oxo-7,8-Dihydroguanine Stability. *Free Radical Biology and Medicine* **2010**, *48*, 89–97, doi:10.1016/j.freeradbiomed.2009.10.029.
3. Matsumoto, Y.; Ogawa, Y.; Yoshida, R.; Shimamori, A.; Kasai, H.; Ohta, H. The Stability of the Oxidative Stress Marker, Urinary 8-Hydroxy-2'- Deoxyguanosine (8-OHdG), When Stored at Room Temperature. *Journal of Occupational Health* **2008**, *50*, 366–372, doi:10.1539/joh.L7144.
4. Wu, C.; Chen, S.-T.; Peng, K.-H.; Cheng, T.-J.; Wu, K.-Y. Concurrent Quantification of Multiple Biomarkers Indicative of Oxidative Stress Status Using Liquid Chromatography-Tandem Mass Spectrometry. *Analytical Biochemistry* **2016**, *512*, 26–35, doi:10.1016/j.ab.2016.07.030.
